# Effect of Temperature on Flavor Compounds and Sensory Characteristics of Maillard Reaction Products Derived from Mushroom Hydrolysate

**DOI:** 10.3390/molecules23020247

**Published:** 2018-01-26

**Authors:** Xiao Chen, Jingyang Yu, Heping Cui, Shuqin Xia, Xiaoming Zhang, Baoru Yang

**Affiliations:** 1 State Key Laboratory of Food Science and Technology, School of Food Science and Technology, Jiangnan University, Wuxi 214122, Jiangsu, China; Jasmine_xiaoc@outlook.com (X.C.); yujingyang@jiangnan.edu.cn (J.Y.); cuihepingdavid@126.com (H.C.); sqxia2006@hotmail.com (S.X.); 2 Food Chemistry and Food Development, Department of Biochemistry, University of Turku, FI-20014 Turku, Finland

**Keywords:** Maillard reaction, mushroom hydrolysate, temperature effect, flavor compounds, sensory attributes, partial least squares regression

## Abstract

Maillard reaction products (MRPs) were prepared from mushroom hydrolysate (MH) by heating with d-xylose and l-cysteine at various temperatures (100 °C–140 °C) for 2 h at a pH of 7.4. The sensory characteristics of MH and MRPs were evaluated by panelists and volatile compounds were analyzed by GC/MS. Additionally, partial least squares regression (PLSR) was performed to analyze the correlation between quantitative sensory characteristics and GC/MS data. GC/MS results revealed that higher reaction temperature resulted in more nitrogen and sulfur containing compounds in MRPs while alcohols, ketones and aldehydes were the major flavor compounds obtained in MH. PLSR results showed that 3-phenylfuran and 2-octylfuran were the compounds responsible for the caramel-like flavor; 1-octen-3-ol, (*E*)-2-octen-1-ol and geranyl acetone were significantly and positively correlated to mushroom-like flavor, whereas, 2-thiophene-carboxaldehyde, 2,5-thiophenedicarboxaldehyde and 3-methylbutanal positively affected MRPs meat-like attribute. Overall, 125 °C was identified as the optimal temperature for preparing MRPs with abundant volatile compounds and favorable sensory characteristics; the concentration of free amino acids and 5′-GMP, which are associated with the umami taste, in MRPs derived under 125 °C were 3 to 4 times higher than those in MH.

## 1. Introduction

The Maillard reaction is a well-known non-enzymatic browning reaction between the amine groups of free amino acids, peptides or proteins and reactive carbonyl groups of reducing sugars under thermal processing and/or food storage conditions [1]. The Maillard reaction is of great importance for the quality of foods and plays a potential role in the production of breads, roasted coffee, vegetables and cooked meat with unique flavor [2]. Nowadays, various findings have demonstrated that the Maillard reaction can confer taste-enhancing (“kokumi”) properties including mouthfulness and continuity characteristics of umami taste to the hydrolyzed proteins [3,4,5]. Song et al. [6] reported that the improved flavor characteristics of soybean protein hydrolysate were related to the cross-linking effect of microbial transglutaminase (MTGase). Moreover, Maillard reaction products (MRPs) not only contributed to the flavor formation, antioxidant and antimicrobial effect, but also played an important role in the improvement of functional properties [7,8,9]. However, the development of flavor-enhancing products based on Maillard reaction is still restricted for several vegetable protein resources, including sunflower [4], soybean [3,6] and corn [2], all of which possess limited sensory characteristics and can hardly meet the growing expectations of the consumers.

Fresh and preserved mushrooms are utilized as raw materials and seasonings all over the world due to their flavor compounds, which can interact with other substances in food and provide customers with desired sensory experience [10]. The fruiting bodies of edible mushrooms are considered as a rich source of protein which can compete with meat; they have low levels of fats (with dominant essential unsaturated fats), functional polysaccharides and nutritious vitamins [11,12]. The taste components of mushrooms, in terms of free amino acids, 5′-ribonucleotides and organic acids have been extensively studied and their equivalent umami concentrations were determined [13,14,15]. Moreover, Dermiki et al. reported that mushroom extracts could be used to enhance the umami taste of food without significantly changing the flavor attributes of final products [16]. This was further proved by Guinard et al. that mushrooms can also be healthy alternatives to meat and could help to reduce the use of sodium (salt) without any decrease in sensory appeal among consumers [17]. The replacement of 80% of the meat by mushrooms has contributed to the sodium reduction up to 25% in terms of the overall impact of the dishes [18]. Therefore, it is desirable to further enhance the flavor and sensory characteristics of mushrooms through the Maillard reaction based on mushroom hydrolysate and provide mushroom MRPs with more opportunities for further use as seasonings in different foods.

On the other hand, different parameters such as temperature, pH, reaction time, and reactants concentration significantly affect the production of MRPs in terms of chemical composition and sensory properties. Thus, different studies have been carried out to determine favorable parameters and precursors in order to enhance the sensory characteristics of MRPs [3,5,19,20]. The reaction temperature is the most important parameter which affects the reaction rate and antioxidant capacity of MRPs [21]; also, the thermal treatment can produce MRPs with carcinogenic compounds, such as acrylamide, furans and furan analogues, all of which have negative health effects and should be taken into consideration [22]. On the other hand, both peptide degradation and peptide cross-linking, which relate to the formation of flavor compounds, are highly affected by the reaction temperature as well. Karangwa et al. studied the temperature effect on sunflower protein MRPs and found that different temperature ranges significantly affected the physicochemical characteristics, the volatile compounds formation and sensory attributes of MRPs [4]. Lan et al. also reported that 100 °C was the critical temperature and above 100 °C peptides degraded quickly in a thermal degradation system [23]. Therefore, it is worthwhile to evaluate and understand the effect of Maillard reaction parameters, especially temperature, on the change of flavor compounds and sensory attributes in order to adjust and improve the characteristic flavor of mushroom MRPs.

Due to the complexity of the Maillard reaction, the study of aromatic composition and the investigation of the relationship between instrumental and sensory descriptive analysis data can provide important information for us to understand the MRPs. Partial least squares regression (PLSR) has been effectively used to explain the correlation between variables by obtaining information from raw data and focusing on a comprehensive evaluation of these information [19]. The correlation between sensory attributes, volatile compounds, peptides molecular weights and sensing system (e-nose) has been studied using PLSR during past few years [4,19,24,25]. Karangwa et al. reported that sulfur containing compounds showed a significant and positive correlation to sensory attributes of Maillard peptides derived from sunflower protein, whereas, nitrogen containing compounds and furans had significant correlation but showed negative effects [4]. Using PLSR, Song et al. found that the beef base with a hydrolysis degree of 29.13% was a desirable precursor for imparting beef-like flavor [19]. Furthermore, it was reported by Yu et al. that the molecular weight distribution of raw peptides had a significant influence on the sensory characteristics of MRPs, and MRPs derived from 1000 to 3000 Da were positively correlated with umami taste [25]. Thus, PLSR can be used to correlate sensory characteristics to aroma compounds in mushroom MRPs, further exploring odorant volatile contributors which can affect the sensory profile of MRPs.

The objective of this study was to evaluate the effect of reaction temperature on flavor characteristics, including the chemical profile of volatile compounds and sensory characteristics of MRPs derived from mushroom hydrolysate (MH). The relationship between volatile compounds and sensory attributes was studied through the PLSR analysis to investigate the key contributors to corresponding sensory characteristics. Furthermore, the contents of free amino acids and 5′-ribonucleotides both in MH and the optimal MRPs were also analyzed and compared to explore the impact of the Maillard reaction on the formation of non-volatile compounds. The information generated through this study may help us adjust the flavor compounds in mushroom MRPs by controlling reaction temperature, further developing the flavor enhancer with favorable sensory characteristics.

## 2. Results and Discussion

### 2.1. Effect of Reaction Temperature on the Volatile Compounds of MRPs

A total of 76 volatiles were identified among MH (the initial hydrolysate without Maillard reaction) and MRPs prepared at different reaction temperatures (100 °C to 140 °C). The concentration of each compound, grouped by chemical family, and the total concentration of each chemical group are reported in Table 1. These chemical groups included 14 alcohols, 13 aldehydes, 11 ketones, eight nitrogen-containing compounds, 16 sulfur-containing compounds, seven furans, two alkenes, two ethers, one ester, one acid and one phenol. 

The free amino acid composition will widely affect the formation of volatile compounds [26]. In this study, different addition ingredients were compared during the pre-test part and cysteine was chosen to be added into MH before Maillard reaction to form nitrogenous and sulfurated heterocyclic compounds, which could provide savory, meaty, caramel and roast flavor as well as continuity taste to the final reaction products [4,6]. These flavor can enrich sensory characteristics of MRPs, further facilitating the application of mushroom MRPs as seasonings in different dishes. On the other hand, cysteine addition in mushroom hydrolysate may effectively reduce the formation of acrylamide, which is widely considered as toxicological compounds mostly formed during the Maillard reaction [27,28].

From Table 1, the main differences between the volatile profiles of MH and MRPs were related to sulfur and nitrogen containing compounds, which were not detected in MH but only in MRPs. In addition, these nitrogenous and sulfurated compounds increased in quantity with raising reaction temperature. MRP140 showed the highest content of sulfur containing compounds (127.40 ng/g) and nitrogenous compounds (22.69 ng/g) compared with other MRPs samples. Some typical volatiles, including 1-furfurylpyrrole, 1-methyl-2-pyrrolidinone, 3-thiophenecarboxaldehyde, 5-methyl-2-thiophenecarboxaldehyde, benzothiazole and 3-methylthiophene, did not appear in MRPs until the temperature increased to 125 °C. Most of these compounds were supposed to exert important effects on the formation of meaty flavor [6]. Additionally, there was only one sulfur containing compound, 2-methyl-5-propyl-thiophene (0.76 ng/g) in MRP100, which was less than half of its concentration in MRP125 (2.17 ng/g). These results might be due to the fact that higher temperature contributes to the thermal degradation of cysteine and carbonyl compounds, forming volatile compounds with sulfur and nitrogen groups in the final products [20]. Furthermore, 2-ethyl-6-methylpyrazine was the major nitrogen containing compound and its concentration increased dramatically from 0.93 ng/g at 120 °C to 8.31 ng/g at 130 °C. The methylpyrazine was only detected after the temperature was raised to 120 °C. These results were consistent with previously reported findings by Tan and Yu [29], that the increased temperature resulted in a more significant increase in the formation of pyrazines and some pyrazines were not formed in their designed systems involving L-ascorbic acid with Asp or Glu until the reaction temperature reached 120 °C.

On the other hand, researchers have reported that the sulfur substituted furans and related disulfides could provide the food with strong meat-like or roast aroma even with low odor threshold value in water [4].

In the present study, the concentration of 3,3′-dithiobis(2-methyl)-furan increased from 0.18 ng/g at 125 °C to 1.14 ng/g at 140 °C and methylfurfuryl disulfide increased from 0.94 ng/g at 110 °C to 5.33 ng/g at 140 °C. This suggests that MRP140 may possess stronger caramel-like attributes compared with other MRPs. Moreover, furan analogues classified as possibly carcinogenic to humans have been considered to be of primary importance for the flavor industry [22]. In our study the total concentration of furan varying from 21.66 ng/g to 71.07 ng/g, the content being significantly lower compared with furan analogues levels in some processed foods for daily use. For example the roasted coffee contains the maximum level of 6500 µg/kg and the average furan level for soups containing meat is 88 µg/kg [30]. It is worth mentioning that soy sauce, used as a common domestic food flavor enhancer, contains average and maximum furan levels of 92 ng/g and 272 ng/g, respectively [31], which is much higher than the level in MRPs prepared in our study.

Alcohols were the most abundant group of volatile compounds detected in all samples. The two alcohol compounds with the highest concentration were 1-octen-3-ol and 2-octen-1-ol. 1-Octen-3-ol was described to possess the typical mushroom-like aroma [32,33], and its content increased with increasing temperature; the highest concentration was obtained in MRP125 (339.26 ng/g) which was increased by 50.90% compared with MH (224.83 ng/g). MRP125 also showed the highest content of (*E*)-2-octen-1-ol with a figure of 21.76 ng/g, while sample MH had the lowest (12.91 ng/g). The group with the second highest concentration of volatile compounds was represented by aldehydes. Significant differences were found among MH and MRPs for aldehyde content. There was an average of 175.40 ng/g for MRPs while the content of aldehyde for MH was only 39.94 ng/g. It was widely reported that aldehydes often possess low thresholds, in the range of a few micrograms per liter water, and thus can play a major role in odor contribution, even if present in low concentrations [34]. Hexanal, which is described to possess a grass-like and leaf-like aroma [19], showed a concentration range varying from 25.56 ng/g in MRP140 to 41.33 ng/g in MRP100. Another main aldehyde was heptanal, which was only detected in MRPs samples and reduced in quantity with increasing temperature. The formation of these volatile compounds were not only related to the Maillard reaction but also be affected by lipid degradation [35]. Autoxidation of lipids leads to the formation of hydroperoxides as primary oxidation products [36,37], which are very unstable, further degrading into various smaller molecules, including alcohols, aldehydes and ketones [38,39].

**Figure 1:**
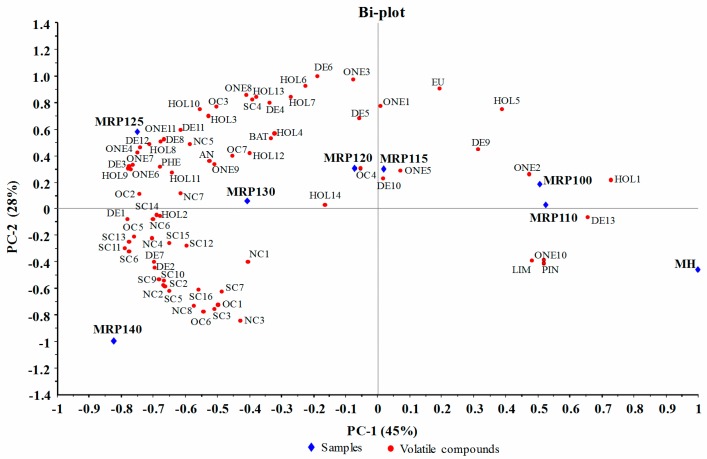
Bi-plot of volatile compounds and samples.

Principal components analysis was conducted using the average content of the compounds reported in Table 1. As shown in the PCA map (Figure 1), Principal Components (PC) 1 and 2 explained 45% and 28% of the variation on the data, respectively. MH, MRP 100, and MRP 110 were located on the right side of the PCA map showing no correlation with most of the volatile compounds. With the increase of the reaction temperature, MRPs showed increasing correlation with volatiles and moved to the left side of the PCA map. Most of the alcohols, ketones, and aldehydes occurred in the left-hand upper quadrant correlated with MRP125 and MRP130; sulfur and nitrogen containing compounds were mostly in the left-hand lower quadrant with more association with MRP140. MRP125 and MRP130 can be grouped together in the PCA bi-plot. These samples had two characteristics in common: (1) they had more types of volatile compounds (Table 1), and (2) volatiles detected in these samples showed higher concentration (Table 1). Overall, PCA map clearly revealed the different volatile composition in MH and seven MRPs; MRPs prepared under high temperature possessed more abundant volatiles profile compared to the substrate (MH) and the MRPs prepared under lower temperatures. The above results showed that higher temperature was favorable for the formation of furans, sulfur and nitrogen containing compounds while the moderate temperature (125 °C), was beneficial to the generation of alcohols, aldehydes and ketones. In addition, significant differences were observed between the composition and the relative content of volatile compounds both in MRPs and MH; this suggested that Maillard reaction can enrich the chemical profile and provide potential flavoring properties (taste and smell) to mushroom hydrolysates.

### 2.2. Sensory Characteristics of Mushroom MRPs and MH Samples

A descriptive analysis for MH and seven MRPs (MRP100-140) was performed and results are presented in Table 2. All samples showed significant differences for all six sensory attributes (caramel-like, mushroom-like, meat-like, continuity, umami and bitterness), which indicated that different reaction temperature can result in various sensory characteristics of final products. Panelists’ results showed no significant effect on all attributes, which suggested minor physiological differences of panelists in perceived intensity when evaluating samples [19]. Moreover, a significant replication effect (*p* < 0.001) was only found for two out of six attributes, namely, caramel-like and mushroom-like; this indicated that the variation based on systematic session in the data was not significant [40]. In addition, no significant interaction was observed between panelists and replication except for caramel-like (*p* < 0.01) and mushroom-like (*p* < 0.05) attributes, which suggested that panelists’ results were reproducible during triplicate tests for each sensory attribute. Furthermore, sample and panelists showed significant interaction for all six attributes (*p* < 0.001) which demonstrated that the panelists scored samples for each sensory attribute inconsistently. The interaction between sample and replication was not significant in all sensory attributes except for the continuity, implying that the other five attributes in all samples showed good repeatability. Generally, these results revealed that the overall sensory characteristics data were acceptable.

The mean intensity value of six attributes and the results from Duncan’s multiple comparison test are shown in Table 3. Panelist assessment revealed that the score of meat-like flavor was higher in MRP140 and was lower in MH. The meat-like flavor might attribute to the higher content of sulfur-containing compounds, such as methylfurfuryl disulfide and 2-acetylthiazole [5]. Referring to the GC/MS results listed in Table 1, MRP140 possessed the highest concentration in methylfurfuryl disulfide (5.33 ng/g) and 2-acetylthiazole (72.43 ng/g) among all MRPs samples. A similar finding has been found by Karangwa et al. [4] when analyzing sensory characteristic of sunflower MRPs prepared at different temperature. On the other hand, though the caramel-like profile was found to have little difference among MRP125 and MRP140, the score of caramel-like attribute showed a general increase trend with raising temperature. This was due to the fact that the high temperature increased sugar caramelization and carbohydrate degradation [41], forming more furans and furanones, which contribute most to the caramel-like characteristic of the products. Moreover, MRP125 presented the strongest mushroom-like attribute and this was consistent with the fact that typical mushroom volatile compounds, including 1-octen-3-ol, (*E*)-2-octen-1-ol, geranyl acetone, etc., detected by GC/MS were the highest in MRP125 compared with other MRPs (Table 1). The highest score of continuity and umami attributes were both obtained in MRP125. The continuity characteristic means a sensation of long-lasting mouthfulness and is related to the formation of Maillard peptides with molecular weight between 1000 and 5000 Da [3,19]. The umami attribute might be associated with the formation and/or the change of non-volatile compounds during the Maillard reaction; these non-volatile compounds include free amino acid, especially aspartic and glutamic amino acids, and 5′-ribonucleotides in terms of 5′-IMP and 5′-GMP. Panelist sensory evaluation results also revealed that the bitterness attribute in MRPs was much lower than that in MH, and the bitterness score decreased significantly with raising reaction temperature. This might be due to the reduction of bitter free amino acids caused by cross-linking between sugars and free amino acids during the Maillard reaction [23]. Another reason might be the loss of low molecular weight peptides which normally act as important active reactants in the Maillard reaction to produce flavor enhancing peptides [3].

### 2.3. Relationship between Volatile Compounds and Sensory Characteristics of Mushroom MRPs

**Figure 2:**
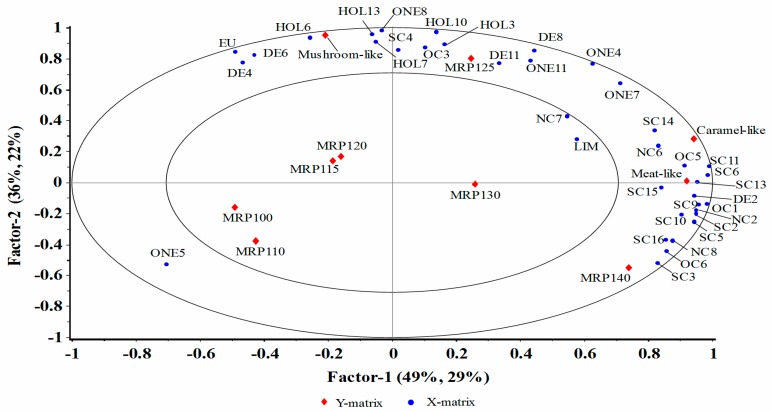
The correlation loadings plot for MRPs samples by partial least squares regression analysis. *X*-matrix = GC/MS analysis; *Y*-matrix = MRPs samples and sensory evaluation data.

In order to improve the sensory profile of mushroom MRPs, it is worthwhile to investigate which aroma compounds have great effects on the MRPs sensory characteristics. PLSR was performed by processing the mean data accumulated from quantitative sensory evaluation of the panelists (Table 3) and the average concentration of volatiles for each MRPs analyzed by GC/MS (Table 1). The *X*-matrix was designated for the mean concentration of volatile compounds shown in Table 1; the *Y*-matrix was set for seven MRPs samples prepared under different reaction temperatures and three sensory attributes, namely caramel-like, mushroom-like and meat-like (Figure 2). The optimal number of Factors in the PLSR model was chosen based on RMSE and explained variances; Three Factors was determined: the explained variance for *X* variables was Factor 1 = 49%, Factor 2 = 36%, Factor 3 = 6% while for *Y* variables, the explained variance of these models was Factor 1, Factor 2 and Factor 3 with 29%, 22% and 12%, respectively. Moreover, only Factor 1 vs. Factor 2 was shown in Figure 2, since further Factors did not provide any predictive improvement in the obtained *Y*-matrix.

As shown in Figure 2, MRP140 and most of the nitrogen and sulfur containing volatile compounds co-varied with two sensory attributes, namely, caramel-like and meat-like, appeared alongside Factor 1 to the right side (positive part) of the plot. Factor 2 was found to be loaded by MRP125 and sensory characteristic of mushroom-like with most of alcohols and aldehydes at the upper part. All *Y* variables except five MRPs and all *X* variables except 2-formylpyrrole (NC7) and D-limonene (LIM) were located between the inner and outer ellipses, which indicated that these variables could be well explained by the PLSR model. 

From Figure 2, several potential correlations were observed between sensory characteristics of the MRPs and volatile compounds detected. Compounds, such as 2-acetylthiazole (SC13), 2-thiophenecarboxaldehyde (SC6), 2-pentylthiazolidine (SC15) and 3-methylbutanal (DE2) were related to meat-like attributes. Furthermore, 2-ethyl-6-methylpyrazine (NC6) and benzothiazole (SC14) and 2-octylfuran (OC5) were correlated to caramel-like attributes. Most of these compounds are nitrogenous and sulfurated heterocyclic compounds and are widely reported as key contributors to the burnt, roasty and meat-like flavors of foods [4,5,19]. In addition, 1-octen-3-ol (HOL6) and 2-octen-1-ol (HOL13), also known as mushroom alcohol, have been described by literatures to be associated with the characteristic flavor of most edible mushrooms [32,33]. In this study, they were indeed related to the mushroom-like attribute in MRPs. Hexanal (DE4) reported to have association with green, fruity [19] and fat or tallow odors [34] was actually related to the mushroom-like attribute in this study. On the other hand, MRP100 and MRP110 occurred in the left-hand lower quadrant while MRP115 and MRP120 grouped in the middle; they are significantly distinguishable from MRP140 and MRP125 which located on the right and upper side of the plot, respectively. Therefore, the different temperature treatments had a significant influence on MRPs sensory characteristics. From Figure 2, MRP125 was associated with the mushroom-like attribute and most of alcohol compounds, while MRP140 correlated with meat-like and caramel-like attributes. These results were in accordance with sensory evaluation score shown in Table 3.

**Figure 3:**
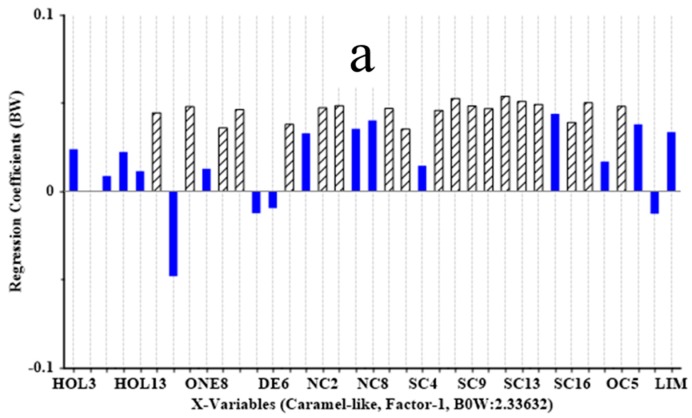

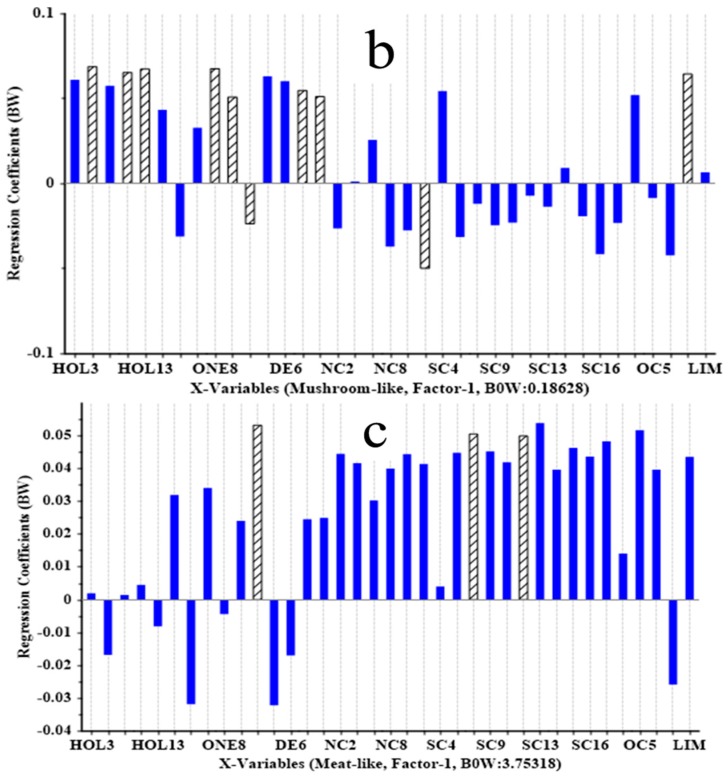
Regression coefficients and significant indications (shown in streaked bars) for sensory attribute variable (**a**) caramel-like (**b**) mushroom-like and (**c**) meat-like analyzed from PLS1 models.

Additionally, PLS1 regression analysis was carried out to further investigate which aroma compounds have the significant contribution to individual sensory attribute of MRPs (Figure 3). The significant variables for each sensory characteristic were inspected by calculating estimated regression coefficients from the jack-knife uncertainty test. Results presented in Figure 3a showed that, except for hexanal (DE4), octanal (DE6), (*E*)-3-octen-2-one (ONE5) and eucalyptol (EU), the other aroma compounds showed positive correlation to caramel-like attribute and among them, ketones including 6-methyl-5-hepten-2-one (ONE4), 2-decanone (ONE7), geranyl acetone (ONE11); two aldehydes containing 3-methylbutanal (DE2) and nonanal (DE8); nitrogen-containing compounds including 1-furfurylpyrrole (NC2) and 2-ethyl-6-methylpyrazine (NC6); most of the sulfur-containing compounds except 2-methyl-5-propylthiophene (SC4) and 2-pentylthiazolidine (SC15); oxygen-containing compounds, namely 3-phenylfuran (OC1) and 2-octylfuran (OC5) had significant positive contribution. A previous study reported that furans can be produced from sugar caramelization and carbohydrate degradation and they contributed to the stronger caramel-like flavor of MRPs [34]. Similarly, 1-octen-3-ol (HOL6), 1-hepten-4-ol (HOL10), (*E*)-2-octen-1-ol (HOL13), nonanal (DE8), benzaldehyde (DE11), (+)-2-bornanone (ONE8), geranyl acetone (ONE11) and eucalyptol (EU) were significantly and positively correlated to mushroom-like flavor (Figure 3b). These compounds were also widely reported to be major contributors to the characteristic odor of mushrooms [33]. Furthermore, most of the sulfur and nitrogen compounds as typical Maillard reaction components showed a positive correlation with meat-like attribute and the impact of 2-thiophenecarboxaldehyde (SC6), 2,5-thiophenedicarboxaldehyde (SC11) and 3-methylbutanal (DE2) was significant (Figure 3c). These sulfur or nitrogen containing compounds mostly formed through cross-linking during the Maillard reaction were reported to have effects on the formation of meaty flavors [6]. Similar findings were reported by Karangwa et al. that 2,5-thiophenedicarboxaldehyde was significantly and positively correlated to meat-like flavor in MRPs derived from the d-xylose and l-cysteine model [4].

Compared with other MRPs, the MRP125 had the highest amounts of 1-octen-3-ol (339.26 ng/g), 1-hepten-4-ol (11.90 ng/g), (*E*)-2-octen-1-ol (21.76 ng/g), nonanal (32.91 ng/g), benzaldehyde (22.49 ng/g), 2-bornanone (43.34 ng/g), geranyl acetone (2.02 ng/g) and eucalyptol (63.71 ng/g) (Table 1); all of them are the key aroma compounds contributing to mushroom-like sensory attributes as analyzed by PLS1. Additionally, the amount of sulfur- and nitrogen-containing compounds in MRP125 was also higher than those in MRP110-120, especially for the key meat-like volatiles, for instance, 2-thiophenecarboxaldehyde (4.55 ng/g), 2,5-thiophenedicarboxaldehyde (1.97 ng/g) and 3-methylbutanal (15.15 ng/g), though slightly lower compared with MRP130 and MRP140. Furthermore, sensory assessment results revealed that MRP125 possessed the highest score for desired sensory characteristics including mushroom-like and umami, while for bitterness, the score was relatively low (Table 3). Hence, the MRP125 was found to be the optimal Maillard reaction product with the desired aspects of both smell and taste.

### 2.4. Comparison of Mushroom Hydrolysate and the Optimal MRPs in Terms of Free Amino Acids and 5′-Ribonucleotides 

**Figure 4:**
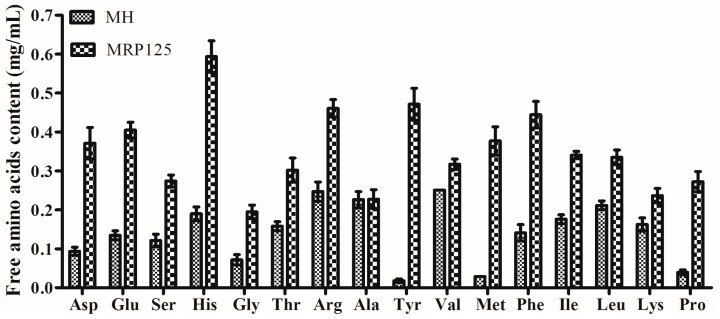
Content of free amino acids in mushroom hydrolysate (MH) and Maillard reaction products prepared at 125 °C (MRP125).

Generally, non-volatile compounds, especially free amino acids, can directly or indirectly influence the sensory perception of MRPs [13]. Free amino acids in MH and MRPs were evaluated in order to study the impact of the Maillard reaction on the formation of non-volatile compounds. The content of free amino acids both in MH and MRP125 are depicted in Figure 4. Sixteen amino acids were detected and each of them was significantly increased (*p* < 0.05) after Maillard reaction. In addition, the total free amino acids content dramatically raised from 2.29 mg/mL in MH to 6.23 mg/mL in MRP125. It is reported that the change of free amino acids in final MRPs is associated with the balance between peptide degradation and free amino acid pyrolysis or cross-linking between peptides [4,23]. The increase of free amino acids content after the Maillard reaction in this study might be attributed to the peptide degradation. This is verified by Karangwa et al., who reported that cysteine addition suppressed the cross-linking of low molecular weight peptides and accelerated the degradation of high molecular weight peptides, forming more free amino acids in MRPs [4]. Moreover, Asp and Glu are generally known to represent the umami-taste amino acids and may reflect the umami properties of final products [16]. For the total concentration of umami-taste amino acids, more than 3-fold increase for MRP125 (0.78 mg/mL) was observed compared with MH (0.25 mg/mL), which indicated that MRP125 possessed better umami taste than MH. These results were in agreement with umami properties obtained from sensory evaluation. Moreover, it was reported that hydrophobic amino acids including Ser, His, Arg, Val, Met, Phe, Ile and Leu were related to bitterness in products, and among them, Arg, His and Met had a stronger bitter perception [16,20]. Though, the concentration of these bitter taste amino acids in MRP125 was 2.89 mg/mL, higher than those in MH (1.26 mg/mL), however, the content of free amino acids related to bitterness in MRP125 was reduced to 46.03% from 54.40% in MH due to the increase of total free amino acids content after Maillard reaction. The high concentration of bitter amino acids does not necessarily result in the bitter taste in food since the overall taste is mostly determined by the balance of various non-volatile compounds other than single component [20]. This could explain why MRP125 had a lower bitterness score than MH when perceived by panelists.

**Figure 5:**
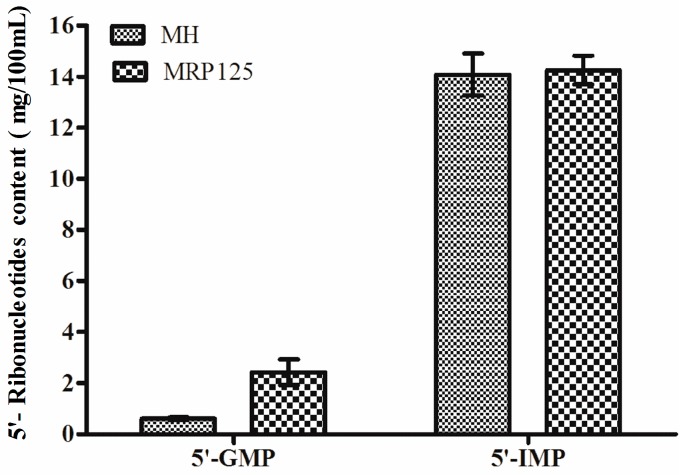
Content of 5′-ribonucleotides in mushroom hydrolysate (MH) and Maillard reaction products prepared at 125 °C (MRP125).

On the other hand, mushrooms are one of the many foods with strong umami taste due to the presence of the 5′-ribonucleotides, particularly 5′-GMP and 5′-IMP. It was reported that the enzyme named 5′-phosphodiesterase broke down RNA in mushrooms into 5′-ribonucleotides during autolysis and the 5′-phosphodiester linkage of 5′-ribonucleotides further splited into flavor compounds by phosphomonoesterases [16]. This process was highly affected by the temperature. Results showed that after Maillard reaction, the concentration of 5′-GMP increased dramatically from 0.60 mg/100 mL in MH to 2.42 mg/100 mL in MRP125 (Figure 5), however there was no significant difference for the concentration of 5′-IMP. In addition, the total content of 5′-ribonucleotides in MRP125 was increased by 13.60% compared to that in MH, which indicated that Maillard reaction could have contributed to the umami taste in the final MRPs. 

## 3. Materials and Methods

### 3.1. Materials

Freeze-dried Finnish mushroom (*Craterellus tubaeformis*) was obtained from Lyotech Oy Co. Ltd. Company (Helsinki, Finland). The aroma compounds in the freeze-dried mushroom were extracted as essential oil by supercritical CO_2_ (FLAVEX Naturextrakte GambH Co. Ltd., Rehlingen, Germany) for commercial purposes and the corresponding residues were used in this study. Cellulase produced by Novozyme Nordisk (Bagsvaerd, Denmark) was purchased from Novo Co., Ltd. (Shanghai, China). Neutral protease was purchased from Longda Bio-Products Co., Ltd. (Yishui, Shandong, China). 1,2-Dichlorobenzene and *n*-alkane mixtures were all chromatography grade and purchased from Sigma-Aldrich Co., Ltd. (Shanghai, China). Authentic standard compounds including 1-octen-3-ol, hexanal, heptanal, pentanal, nonanal, benzaldehyde, octanal, and 6-methyl-5-hepten-2-one were obtained from Sigma-Aldrich Co., Ltd. The other chemicals used were of analytical grade and were obtained from Shanghai Chemical Reagent Co. Ltd. (Shanghai, China).

### 3.2. Methods

#### 3.2.1. Preparation of Mushroom Hydrolysate

Milled dry mushroom samples (10 g, containing 2.1 g of crude protein) were mixed with distilled water in a ratio of 1:14 (*w*/*v*). The mixtures were pre-treated at 85 °C for 30 min in order to destroy the protein tertiary structure. After cooling the mixture to 0 °C using ice bath, the enzymatic hydrolysis process was conducted in two steps. In the first step, the pH of the mixture was adjusted to 5.0 using HCl (2 mol/L), then the cellulase was added with a cellulase/sample ratio of 2 g/100 g while the temperature was set at 55 °C for 2 h. In the second step the pH was adjusted to 7.0 using 2 mol/L sodium hydroxide (NaOH), then neutral protease was added with a neutral protease/sample protein ratio of 3 g/100 g (the protein content in sample was measured by the Kjeldahl method); the mixture was further digested for another 2 h at 55 °C. All the parameters were obtained from the process optimization. Furthermore, the hydrolysate was heated at 100 °C for 15 min to inactivate the enzymes and then centrifuged at 10,000 rpm for 25 min at 4 °C (RXII Series, Hitachi, Tokyo, Japan). The final supernatant (mushroom hydrolysate, MH) was collected, sealed in tubes and stored at −20 °C until use.

#### 3.2.2. Preparation of Maillard Reaction Products (MRPs)

d-Xylose (0.10 g) and l-cysteine (0.19 g) were dissolved into 30 mL MH. The pH of the mixture was adjusted to 7.4 with 2 mol/L HCl and/or 2 mol/L NaOH. Then the mixture was transferred into a Pyrex vial (50 mL), fitted with magnetic stirrer, sealed tightly and heated in a thermostatic oil bath for 120 min with the temperature range from 100 °C to 140 °C. The obtained products after heating were termed ‘Maillard reaction products (MRPs)’ and coded as follows: MRP100 to MRP140. MRPs were immediately cooled into ice water in order to terminate the reaction and were stored at −20 °C till further use. 

#### 3.2.3. Headspace Solid Phase Micro-Extraction/Gas Chromatography/Mass Spectrometry (HS-SPME/GC/MS) Analysis

The volatile compounds of mushroom MRPs were analyzed according to the method of Huang et al. [24] with slight modifications. Briefly, 3 g of each MRPs sample with an internal standard (0.055 µg/µL of 1,2-dichlorobenzene in methanol), were placed into a 15 mL glass vial sealed with PTFE/BYTL septum. The vial was left for 30 min at 50 °C with an SPME-fiber (75 µm, carboxen/poly-dimethysiloxane) to allow equilibration of volatiles in the headspace. The injection was conducted in a splitless mode for 3 min at 250 °C. Separation of volatiles was carried out on a DB-WAX capillary column (30 m × 0.25 µm × 0.25 µm, J&W Scientific, Folsom, CA, USA) with the temperature program as follow: 40 °C for 3 min, then raised to 80 °C at 5 °C min^−1^, raised to 160 °C at 10 °C min^−1^ and held for 0.5 min, raised to 175 °C at 2 °C min^−1^, raised to 230 °C at 10 °C min^−1^, and finally held at 230 °C for 7 min. A mass spectrometric detector operated in the electron impact mode with an energy voltage of 70 eV and emission current of 35 mA was used. The detector was set at a scanning range of 35 to 450 m/z at a rate of 4.45 scans s^−1^.

Volatile compounds identification was performed by comparing mass spectral data of samples with those of the Wiley 6.0 (Wiley, New York, NY, USA) library and the NIST 98 (National Institute of Standards and Technology, Gaithersburg, MD, USA). Kovats indices (KIs) were calculated using an *n*-alkane series (C_5_–C_40_) under the same chromatographic program as the samples and compared with available literature data. Approximate quantities of the volatile compounds were estimated by comparison of peak areas with that of the internal standard. The peak areas were obtained from the total ion chromatograms. The quantitative formula was as follows:

(1) $$W_{x}=f^{\prime }\times \frac{A_{x}*m_{is}}{A_{is}}/m$$

where $W_{x}$ is the concentration (ng/g) of compound *x*, $f^{\prime }$ is a relative correction factor, assumed to be 1, $A_{x}$ is the peak area of compound *x*, $A_{is}$ is the peak area of internal standard, $m_{is}$ is the mass of internal standard, m is the mass of sample.

#### 3.2.4. Determination of 5′-Ribonucleotides

The contents of 5′-ribonucleotides in the MRPs were determined by high performance liquid chromatography. Agilent liquid chromatograph 1100 (Agilent Technologies, Palo Alto, CA, USA) equipped with 2487 UV detector was used for this experiment. The column used was Diamonsil C18 (4.6 mm × 250 µm × 5 µm, J&W Scientific). The mobile phase consisting of methyl alcohol/water/phosphoric acid (5/95/0.5, *v*/*v*/*v*) was delivered at a flow rate of 0.8 mL/min. The column temperature was 30 °C and 10 µL of sample was injected into the HPLC system. A standard curve was obtained from the following standards from Sigma: 5′-GMP with 0.21 mg/mL and 5′-IMP with 0.25 mg/mL. The results were obtained using UV detector at 254 nm, and the data analysis was performed using chromatography software.

#### 3.2.5. Determination of Free Amino Acids

Free amino acids in the samples were analyzed using an Agilent 1100 liquid chromatography system (Agilent Technologies) equipped with a UV detector operated at 338 nm and a column of ODS Hypersil (250 mm × 4.6 mm) at 40 °C. The mobile phase consisted of 20 mM sodium acetate and 1:2 (*v*/*v*) methanol-acetonitrile and delivered at a flow rate of 1 mL/min. For free amino acids content calculation, a calibration curve was obtained with standard amino acid mixture (Sigma Chemical Co., St. Louis, MO, USA) and qualitative analysis was made on the basis of retention time and peak area of standard compounds. 

For the sample pre-treatment part, an equivalent volume of trichloroacetic acid (TCA) was added to the sample to precipitate peptides and/or proteins. The solution was filtered through a filter paper and the filtrate was centrifuged at 10,000 rpm for 15 min. The supernatant was collected and stored at 4 °C before injection.

#### 3.2.6. Sensory Evaluation

Sensory analysis was applied to evaluate sensory characteristics of mushroom hydrolysate (MH) and mushroom MRPs at different reaction temperature (MRP100-MRP140), based on the method proposed by different researchers with slight modifications [3,24]. The well-trained panel was composed of fifteen members (eight females and seven males) with the age range of 23–49 years. All panelists had the experience in sensory profiles of various food samples and had received training in the description of sensory characteristics more than twenty hours. The sensory evaluation took place in a sensory laboratory complied with international standards for test rooms. Four specific training sessions were performed. In the first session, all panelists were trained for an additional three hours and made a mutual consensus (50% of the assessment scale) on scoring. In the second and third session, the panelists discussed MRP flavor characteristics for sensory attributes, defined descriptive terms including mushroom-like, meat-like, caramel-like, continuity, umami and bitterness and further determined appropriate reference solutions. Finally, the test samples were evaluated in triplicate using a scale of 1–9, where a score of 4 was given to the control sample. The reference materials were as follows: mushroom-like (5 g dry mushroom powder heated with 50 mL water at 100 °C for 15 min, filtered); meat-like (5 g of heated defatted brisket meat in 200 mL water, filtered); caramel (2.5 g of burning white sugar in 80 mL water), umami (0.5% (*w*/*v*) monosodium glutamate) and continuity (0.5% (*w*/*v*) monosodium glutamate with dextrin DE 8–10); bitterness (prepared from 4 mM caffeine).

The sample solution (1.0%, *w*/*w*) was individually dissolved in a salty soup consisted of 0.5% (*w*/*w*) salt. Then, 60 mL sample and 60 mL reference solutions were heated and kept at 40 °C in a water bath to avoid temperature differences that could influence the assessment. For testing, samples were coded with three-digit numbers, served in a randomized order and tested at the same time in separated sensory booths at 25 ± 2 °C. The assessment of each samples was done in triplicate by fifteen panelists, and the average of all the panelists’ scores was calculated for sensory characteristics of each sample.

#### 3.2.7. Statistical Analysis

Statistical analysis was performed using Microsoft Excel 2010 (Microsoft, Redmond, WA, USA) and SPSS 19.0 (IBM, Armonk, NY USA). The correlations between volatile compounds of MRPs and corresponding sensory attribute responses were analyzed by PLS1, and PLS2 was applied to illustrate correlations among aroma compounds and sensory attributes datasets. The PLSR analysis was achieved using the Unscrambler software version *X* 10.4 (CAMO ASA, Oslo, Norway), as reported by Song et al. [19]. One way analysis of variance (ANOVA) was used to compare means with Tukey multiple range tests for post-hoc analysis. *p* < 0.05, *p* < 0.01 and *p* < 0.001 were considered significant. Each temperature treatment was performed in triplicate and each experiment was analyzed one time according to the method described above; the results reported in this work were the means of triplicate experiments.

## 4. Conclusions

Temperature effects on the formation of flavor compounds and related sensory attributes for MRPs derived from mushroom hydrolysate were successfully demonstrated. GC/MS analysis revealed that more contents and numbers of sulfur- and nitrogen-containing compounds were formed with the increase of the temperature; a relatively higher temperature (125 °C) was beneficial to the formation of typical mushroom compounds including 1-octen-3-ol, 2-octen-1-ol and nonanal. Furthermore, PLSR analysis correlated volatile compounds with sensory attributes of MRPs prepared under different reaction temperatures and showed that volatiles such as 3-phenylfuran and 2-octylfuran were the key compounds for caramel-like flavor; 1-octen-3-ol, (*E*)-2-octen-1-ol, nonanal, benzaldehyde, (+)-2-bornanone and geranyl acetone were significantly and positively correlated to mushroom-like flavor, while 2-thiophenecarboxaldehyde, 2,5-thiophenedicarboxaldehyde and 3-methylbutanal were identified as possible key volatiles that contributed to the meat-like attributes of MRPs. Furthermore, the comparison between MRP125 (with favorable sensory characteristics) and its substrate (MH) in terms of non-volatile compounds revealed that the Maillard reaction was favorable to increase the concentration of total free amino acids and 5′-GMP, which have close relationship with umami taste. These indicated the potential of the Maillard reaction for increasing the umami taste and reducing the bitterness properties of MH, which could be beneficial and useful for the food and flavor industries.

## Figures and Tables

**Table 1:** Volatile compounds detected in MH and MRPs.

Compounds	Relative Content ng/g										
	MRI ^a^	KI ^b^	ID	MH	MRP100	MRP110	MRP115	MRP120	MRP125	MRP130	MRP140
**Alcohols**											
1-Pentanol	1260	1271^N^	HOL1	0.42 ± 0.02	0.45 ± 0.03	0.33 ± 0.03	0.37 ± 0.03	0.35 ± 0.03	ND	ND	ND
3-Methyl-2-buten-1-ol	1321	1323^N^	HOL2	ND	0.71 ± 0.02	ND	2.04 ± 0.15	1.17 ± 0.06	1.36 ± 0.06	1.44 ± 0.06	2.13 ± 0.11
1-Hexanol	1324	1347^N^	HOL3^PL^	0.61 ± 0.07	1.17 ± 0.07	0.95 ± 0.09	1.04 ± 0.09	1.19 ± 0.07	1.85 ± 0.09	1.17 ± 0.06	0.96 ± 0.07
Borneol	1658	1675^N^	HOL4	ND	ND	ND	ND	3.92 ± 0.10	3.83 ± 0.12	ND	ND
3-Octanol	1394	1394^N^	HOL5	1.98 ± 0.06	2.7 ± 0.21	2.57 ± 0.06	3.05 ± 0.12	2.73 ± 0.10	1.76 ± 0.08	2.62 ± 0.11	0.66 ± 0.07
1-Octen-3-ol	1454	1456^N,STD^	HOL6^PL^	224.83 ± 11.43	284.53 ± 6.04	235.95 ± 13.28	286.51 ± 6.95	288.35 ± 6.06	339.26 ± 2.58	250.38 ± 3.13	211.23 ± 2.12
2,6-Dimethyl-7-octen-2-ol	1463	1474^N^	HOL7^PL^	2.30 ± 0.08	2.64 ± 0.11	2.05 ± 0.12	3.29 ± 0.25	2.74 ± 0.12	3.58 ± 0.21	3.07 ± 0.08	1.87 ± 0.06
1-Octanol	1546	1561^N^	HOL8	2.65 ± 0.06	4.46 ± 0.14	3.61 ± 0.13	5.29 ± 0.16	4.75 ± 0.10	7.08 ± 0.09	5.42 ± 0.10	5.04 ± 0.09
(*R*)-Linalool	1536	1552^N^	HOL9	ND	2.92 ± 0.21	3.15 ± 0.14	7.26 ± 0.11	6.70 ± 0.12	14.27 ± 0.42	8.63 ± 0.11	9.11 ± 0.17
1-Hepten-4-ol	1558	1585^N^	HOL10^PL^	5.26 ± 0.14	8.44 ± 0.14	7.30 ± 0.12	9.07 ± 0.48	9.10 ± 0.16	11.90 ± 1.88	8.60 ± 0.11	7.53 ± 0.12
Terpinen-4-ol	1595	1594^N^	HOL11	ND	ND	0.71 ± 0.07	1.04 ± 0.11	1.25 ± 0.12	1.09 ± 0.18	1.22 ± 0.05	0.94 ± 0.06
(*Z*)-2-Octen-1-ol	1564	1547^N^	HOL12	ND	3.07 ± 0.17	3.08 ± 0.14	4.65 ± 0.69	16.77 ± 1.95	8.35 ± 1.1	5.84 ± 0.11	5.28 ± 0.04
(*E*)-2-Octen-1-ol	1613	1603^N^	HOL13^PL^	12.91 ± 1.54	17.48 ± 0.67	13.46 ± 1.26	17.29 ± 1.27	18.31 ± 1.52	21.76 ± 1.35	16.27±0.06	13.55 ± 0.55
2,6-Dimethyl-3,7-octadiene-2,6-diol	1985	1969^N^	HOL14	5.49 ± 0.57	6.06 ± 0.15	4.56 ± 0.72	5.48 ± 0.65	5.10 ± 0.23	6.22 ± 0.45	4.55 ± 0.15	5.76 ± 0.12
				256.45 ± 13.97	334.65 ± 1.70	277.72 ± 16.14	346.39 ± 11.08	362.44 ± 10.73	422.31 ± 8.61	309.47 ± 4.12	264.06 ± 3.50
**Ketones**											
3-Octanone	1244	1240^N^	ONE1	1.22 ± 0.1	1.33 ± 0.09	1.13 ± 0.19	0.84 ± 0.09	1.01 ± 0.07	2.31 ± 0.18	1.65 ± 0.08	ND
1-Octen-3-one	1287	1280^N^	ONE2	0.97 ± 0.15	0.53 ± 0.06	0.59 ± 0.06	0.68 ± 0.08	ND	0.68 ± 0.06	0.45 ± 0.07	ND
2-Methyl-3-octanone	1310	1322^N^	ONE3	ND	6.06 ± 0.35	6.02 ± 0.14	6.36 ± 0.14	5.74 ± 0.14	6.88 ± 0.09	4.84 ± 0.12	ND
6-Methyl-5-hepten-2-one	1330	1339^N, STD^	ONE4^PL^	2.99 ± 0.14	4.52 ± 0.11	4.17 ± 0.17	5.62 ± 0.41	5.46 ± 0.15	7.21 ± 0.20	5.48 ± 0.16	5.66 ± 0.20
(*E*)-3-Octen-2-one	1338	1396^N^	ONE5^PL^	2.42 ± 0.10	7.04 ± 0.09	6.64 ± 0.19	6.48 ± 0.10	5.53 ± 0.32	3.08 ± 0.15	6.14 ± 0.21	4.52 ± 0.15
5-Ethyl-6-undecanone	1399	1429^N^	ONE6	ND	16.06 ± 0.28	14.45 ± 1.10	16.25 ± 0.67	21.16 ± 2.32	35.41 ± 2.11	28.57 ± 1.98	26.17 ± 1.06
2-Decanone	1487	1493^N^	ONE7 ^PL^	3.47 ± 0.18	18.24 ± 0.81	16.50 ± 0.56	22.40 ± 1.42	21.9 ± 1.16	44.67 ± 2.49	30.48 ± 1.17	30.12 ± 1.04
(+)-2-Bornanone	1505	1528^N^	ONE8^PL^	25.51 ± 2.54	33.91 ± 1.23	27.62 ± 2.62	35.57 ± 1.12	35.84 ± 2.68	43.34 ± 1.45	33.10 ± 2.74	27.30 ± 0.84
2-Undecanone	1589	1592^N^	ONE9	ND	2.04 ± 0.13	1.80 ± 0.16	3.31 ± 0.26	ND	3.91 ± 0.14	3.40 ± 0.19	2.23 ± 0.10
Carvone	1720	1728^N^	ONE10	0.41 ± 0.08	ND	ND	ND	ND	ND	ND	ND
Geranyl acetone	1854	1858^N^	ONE11 ^PL^	0.34 ± 0.02	1.15 ± 0.14	1.08 ± 0.16	1.66 ± 0.12	1.83 ± 0.12	2.02 ± 0.11	1.42 ± 0.04	1.50 ± 0.11
				37.33 ± 3.3	90.86 ± 3.28	80.02 ± 5.34	99.17 ± 4.40	98.46 ± 6.96	149.5 ± 6.98	115.53 ± 6.75	97.51 ± 3.50
**Aldehydes**											
Butanal	865	867^N^	DE1	ND	1.32 ± 0.09	1.35 ± 0.13	1.41 ± 0.15	1.76 ± 0.08	2.50 ± 0.16	1.80 ± 0.06	3.05 ± 0.12
3-Methylbutanal	896	900^N^	DE2^PL^	10.46 ± 0.99	8.72 ± 0.11	10.02 ± 0.76	15.51 ± 1.42	11.62 ± 1.25	15.15 ± 0.20	17.04 ± 0.25	21.49 ± 1.25
Pentanal	975	979^N,STD^	DE3	1.35 ± 0.05	2.16 ± 0.15	2.16 ± 0.17	2.56 ± 0.13	2.89 ± 0.14	3.94 ± 0.10	2.86 ± 0.07	3.12 ± 0.12
Hexanal	1077	1078^N,STD^	DE4^PL^	12.05 ± 0.15	41.33 ± 1.59	32.62 ± 1.95	35.35 ± 1.92	38.16 ± 2.22	45.04 ± 1.23	30.17 ± 1.14	25.56 ± 1.96
Heptanal	1171	1183^N,STD^	DE5	ND	61.22 ± 2.64	55.39 ± 1.72	54.41 ± 1.28	61.82 ± 2.18	35.30 ± 1.35	47.46 ± 2.00	21.68 ± 1.75
Octanal	1278	1291^N,STD^	DE6^PL^	1.77 ± 0.05	10.05 ± 1.56	9.78 ± 1.35	12.33 ± 1.07	11.6 ± 1.15	15.93 ± 1.69	12.70 ± 0.79	ND
(*E*)-2-Heptenal	1287	1318^N^	DE7	ND	7.50 ± 1.01	4.70 ± 0.48	9.31 ± 1.15	5.75 ± 0.1	11.61 ± 1.77	8.63 ± 0.75	21.76 ± 0.62
Nonanal	1384	1396^N,STD^	DE8 ^PL^	5.44 ± 0.06	21.67 ± 1.59	21.64 ± 1.6	26.86 ± 1.31	28.37 ± 1.87	32.91 ± 2.86	25.52 ± 1.5	25.19 ± 1.05
(*E*)-2-Octenal	1420	1427^N^	DE9	ND	4.00 ±0.16	1.67 ± 0.09	2.94 ± 0.11	2.55 ± 0.12	ND	ND	ND
Decanal	1486	1498^N^	DE10	ND	ND	1.54 ± 0.06	1.79 ± 0.14	ND	ND	1.86 ± 0.20	ND
Benzaldehyde	1513	1515^N,STD^	DE11^PL^	6.03 ± 0.13	9.52 ± 0.97	13.16 ± 0.24	17.66 ± 0.83	21.47 ± 0.83	22.49 ± 2.92	18.26 ± 0.53	14.01 ± 0.18
Benzeneacetaldehyde	1635	1640^N^	DE12	1.31 ± 0.06	5.19 ± 0.67	4.54 ± 0.30	6.46 ± 0.92	7.43 ± 0.47	10.55 ± 0.88	7.22 ± 0.29	7.13 ± 0.64
2-Butyl-2-octenal	1662	1653^N^	DE13	1.53 ± 0.10	2.08 ± 0.15	2.52 ± 0.24	ND	ND	ND	ND	ND
				39.94 ± 1.59	174.77 ±10.68	161.09 ± 9.10	186.58 ± 10.44	193.41 ± 10.39	195.44 ± 13.15	173.51 ± 7.58	142.99 ± 7.69
**Nitrogen-Containing Compounds**											
2-Pentylpyridine	1570	1554^N^	NC1	ND	ND	ND	2.41 ± 0.34	ND	ND	1.44 ± 0.07	2.26 ± 0.08
1-Furfurylpyrrole	1792	1820	NC2^PL^	ND	ND	ND	ND	ND	1.03 ± 0.1	0.94 ± 0.06	2.23 ± 0.14
Ethylpyrazine	1329	1323^N^	NC3	ND	ND	ND	ND	ND	ND	ND	0.56 ± 0.08
Methylpyrazine	1264	1263^N^	NC4	ND	ND	ND	ND	0.93 ± 0.1	0.83 ± 0.09	1.15 ± 0.11	1.26 ± 0.03
3-Ethyl-2,5-dimethylpyrazine	1446	1447^N^	NC5	ND	ND	1.7 ± 0.13	3.22 ± 0.14	1.41 ± 0.13	6.69 ± 0.18	5.24 ± 0.12	1.71 ± 0.09
2-Ethyl-6-methylpyrazine	1369	1363^N^	NC6^PL^	ND	0.65 ± 0.10	0.7 ± 0.05	0.67 ± 0.08	0.93 ± 0.12	7.50 ± 0.15	8.31 ± 0.30	6.43 ± 0.14
2-Formylpyrrole	1716	1711^F^	NC7^PL^	ND	ND	ND	2.56 ± 0.08	2.91 ± 0.13	2.77 ± 0.15	0.56 ± 0.07	2.73 ± 0.09
1-Methyl-2-pyrrolidinone	1649	1646	NC8^PL^	ND	ND	ND	ND	ND	1.31 ± 0.11	1.05 ± 0.09	5.52 ± 0.35
				0 ± 0	0.65 ± 0.10	2.4 ± 0.18	8.85 ± 0.64	6.18 ± 0.48	20.12 ± 0.78	18.68 ± 0.82	22.69 ± 0.98
**Sulfur-Containing Compounds**											
3-Methyl-2-thiophenecarboxaldehyde	1770	1765^N^	SC1	ND	ND	ND	ND	ND	ND	ND	2.26 ± 0.09
3-Methylthiophene	1082	1106^N^	SC2^PL^	ND	ND	ND	ND	ND	3.34 ± 0.11	3.14 ± 0.06	7.62 ± 0.11
2-Propylthiophene	1227	1238^N^	SC3^PL^	ND	ND	ND	ND	ND	ND	0.50 ± 0.03	0.88 ± 0.11
2-Methyl-5-propylthiophene	1301	1314^N^	SC4^PL^	ND	0.76 ± 0.08	0.68 ± 0.04	1.00 ± 0.08	0.78 ± 0.07	2.17 ± 0.09	1.66 ± 0.07	ND
3-Thiophenecarboxaldehyde	1668	1666^N^	SC5^PL^	ND	ND	ND	ND	ND	1.51 ± 0.08	1.82 ± 0.10	4.03 ± 0.12
2-Thiophenecarboxaldehyde	1687	1678^N^	SC6^PL^	ND	ND	0.75 ± 0.11	1.86 ± 0.10	1.24 ± 0.13	4.55 ± 0.13	3.67 ± 0.12	6.35 ± 0.09
Methyl furfuryl disulfide	1721	1721^N^	SC7	ND	ND	0.94 ± 0.09	ND	2.94 ± 0.12	ND	3.15 ± 0.13	5.33 ± 0.30
3-Methyl-2-Thiophenecarboxaldehyde	1770	1765^N^	SC8	ND	ND	ND	ND	ND	ND	ND	1.26 ± 0.08
5-Methyl-2-thiophenecarboxaldehyde	1800	1785^N^	SC9^PL^	ND	ND	ND	ND	ND	4.83 ± 0.10	4.82 ± 0.11	9.59 ± 0.26
Thieno(2,3-b)thiophene	1857	1843^F^	SC10^PL^	ND	ND	ND	ND	1.71 ± 0.08	1.46 ± 0.07	1.61 ± 0.11	4.09 ± 0.14
2,5-Thiophenedicarboxaldehyde	1907	1833^N^	SC11^PL^	ND	ND	ND	0.87 ± 0.07	0.94 ± 0.09	1.97 ± 0.05	1.58 ± 0.13	2.84 ± 0.08
Thiazole	1244	1265^N^	SC12	ND	ND	0.49 ± 0.02	0.46 ± 0.05	3.40 ± 0.12	3.08 ± 0.14	ND	4.73 ± 0.41
2-Acetylthiazole	1641	1667^N^	SC13^PL^	ND	ND	15.65 ± 1.24	35.63 ± 2.25	38.19 ± 1.85	44.09 ± 1.90	52.13 ± 6.07	72.43 ± 1.22
Benzothiazole	1959	1968^N^	SC14^PL^	ND	ND	ND	ND	ND	2.04 ± 0.11	1.64 ± 0.25	1.44 ± 0.09
2-Pentylthiazolidine	1828	1838^N^	SC15^PL^	ND	ND	ND	0.85 ± 0.09	0.94 ± 0.09	1.85 ± 0.06	4.58 ± 0.09	3.40 ± 0.04
3,3′-Dithiobis(2-methyl)-furan	2120	2124^N^	SC16^PL^	ND	ND	ND	ND	ND	0.18 ± 0.01	0.95 ± 0.09	1.14 ± 0.08
				0 ± 0	0.76 ± 0.08	18.51 ± 1.50	40.67 ± 2.64	50.14 ± 2.55	71.07 ± 2.84	81.26 ± 7.35	127.40 ± 3.22
**Furans**											
3-Phenylfuran	1849	1872^N^	OC1^PL^	4.45 ± 0.15	ND	ND	1.35 ± 0.11	2.11 ± 0.10	4.23 ± 0.07	4.57 ± 0.07	9.21 ± 0.22
2-Butylfuran	1122	1122^N^	OC2	ND	ND	ND	0.47 ± 0.06	0.28 ± 0.07	0.52 ± 0.04	0.53 ± 0.06	0.47 ± 0.06
2-Pentylfuran	1216	1235^N^	OC3^PL^	ND	21.66 ± 1.52	22.22 ± 1.63	48.11 ± 1.56	30.46 ± 1.80	56.16 ± 2.17	44.66 ± 4.45	15.37 ± 0.65
2-Heptylfuran	1425	1429^N^	OC4	5.64 ± 0.24	ND	4.38 ± 0.12	5.51 ± 0.43	6.33 ± 0.50	5.94 ± 0.10	4.28 ± 0.36	2.64 ± 0.08
2-Octylfuran	1509	1519^N^	OC5^PL^	ND	ND	ND	2.08 ± 0.21	1.45 ± 0.07	1.98 ± 0.16	2.19 ± 0.18	3.20 ± 0.05
3-Furaldehyde	1457	1455^N^	OC6^PL^	ND	ND	ND	ND	ND	1.74 ± 0.09	2.48 ± 0.32	11.56 ± 1.36
2(5*H*)-Furanone	1743	1745^N^	OC7	ND	ND	ND	ND	ND	0.50 ± 0.03	0.54 ± 0.06	ND
				10.09 ± 0.39	21.66 ± 1.52	26.60 ± 1.75	57.52 ± 2.37	40.63 ± 2.54	71.07 ± 2.66	59.25 ± 5.50	42.45 ± 2.42
**Others**											
Anethole	1823	1809^N^	AN	0.47 ± 0.06	1.13 ± 0.11	0.63 ± 0.09	1.59 ± 0.13	1.42 ± 0.06	1.15 ± 0.15	1.33 ± 0.21	1.21 ± 0.12
Eucalyptol	1195	1211^N^	EU^PL^	48.40 ± 2.26	50.95 ± 1.47	40.27 ± 1.26	48.34 ± 0.91	47.89 ± 2.54	63.71 ± 1.36	44.07 ± 3.08	15.45 ± 1.37
α-Pinene	1040	1043^N^	PIN	2.43 ± 0.13	ND	ND	ND	ND	ND	ND	ND
D-Limonene	1168	1189^N^	LIM^PL^	84.30 ± 3.59	ND	1.75 ± 0.07	7.07 ± 0.14	5.52 ± 0.54	4.54 ± 0.27	6.67 ± 0.33	5.26 ± 0.13
Bornyl acetate	1573	1567^N^	BAT	1.35 ± 0.09	1.61 ± 0.15	1.40 ± 0.03	ND	3.50 ± 0.48	4.85 ± 0.13	2.35 ± 0.22	1.02 ± 0.11
Octanoic acid	2064	2070^N^	OTA	ND	ND	ND	ND	ND	ND	ND	1.40 ± 0.04
Phenol	1995	2008^N^	PHE	ND	ND	0.60 ± 0.06	0.68 ± 0.07	0.98 ± 0.11	1.08 ± 0.07	0.85 ± 0.13	0.76 ± 0.06
				136.95 ± 6.13	53.69 ± 1.73	44.65 ± 1.51	57.68 ± 1.25	59.31 ± 3.73	75.33 ± 1.98	55.27 ± 3.97	25.10 ± 1.83

^a^ MRI means the Kovats index which were determined by a series of hydrocarbons (C8–C40) on the column of DB-WAX as described in Section 3.2.3; ^b^ KI denotes the Kovats index reference from NIST Standard Reference Database, by which the compositions were determined on a polar (PE/DB-WAX) column run under similar GC-MS conditions; The identification is indicated by the following symbols: (N) mass spectrum compared with NIST98 and Wiley 6.0; (F) MRI compared with reference; (STD) compared with authentic standard. All GC peak areas were quantified relative to the internal standard (1,2-dichlorobenzene); ND: not detected, MRP100–140: Maillard reaction products prepared at the temperature of 100–140 °C, PL: code representing volatile compounds used in the PLSR analysis. HOL: alcohols, DE: aldehydes, ONE: ketones, NC: nitrogen-containing compounds, SC: sulfur-containing compounds, OC: furans, AN: anethole; EU: eucalyptol, PIN: α-pinene, LIM: d-Limonene, BAT: bornyl acetate, OTA: octanoic acid, PIN: phenol.

**Table 2:** Analyses of variance for the main effects and their interactions for each of the six attributes in sensory evaluation of MH and MRP100–140.

Sensory Attributes	*F*-Values					
	Sample (S)	Panelist (P)	Replication (R)	S × P	P × R	S × R
	(df = 8)	(df = 15)	(df = 3)	(df = 120)	(df = 45)	(df = 24)
Caramel-like	413.52 ^***^	0.75	18.39 ^***^	1.72 ^***^	2.16 ^**^	1.16
Mushroom-like	197.20 ^***^	1.17	11.09 ^***^	2.57 ^***^	1.58 ^*^	0.65
Meat-like	455.16 ^***^	1.42	1.92	2.10 ^***^	1.35	1.14
Continuity	166.60 ^***^	0.92	0.56	1.91 ^***^	1.48	2.93 ^****^
Umami	212.44 ^***^	1.09	3.23	2.24 ^***^	1.07	1.50
Bitterness	404.31 ^***^	1.27	4.79 ^*^	3.22 ^***^	1.48	0.70

* Significant at *p* < 0.05; ** Significant at *p* < 0.01; *** Significant at *p* < 0.001.

**Table 3:** The mean intensity values of six attributes for MH and seven MRPs samples in sensory evaluation.

Samples	Mean Score					
	Caramel-Like	Mushroom-Like	Meat-Like	Continuity	Umami	Bitterness
MH	1.5 ^a^	3.6 ^a^	1.1 ^a^	3.2 ^b^	1.5 ^a^	8.1 ^f^
MRP100	3.3 ^b^	5.1 ^b^	3.5 ^b^	2.0 ^a^	2.3 ^b^	6.4 ^e^
MRP110	3.6 ^b,c^	4.6 ^b^	4.7 ^c^	3.6 ^b^	2.5 ^b^	6.7 ^e^
MRP115	4.1 ^c^	5.5 ^c^	5.6 ^d^	5.5 ^d^	3.5 ^c^	5.5 ^d^
MRP120	4.7 ^d^	6.4 ^d^	5.0 ^c,d^	5.0 ^c,d^	3.9 ^c^	4.8 ^c^
MRP125	5.9 ^f^	7.7 ^e^	5.8 ^d,e^	6.4 ^e^	4.9 ^d,e^	4.7 ^c^
MRP130	5.3 ^e^	4.8 ^b,c^	6.3 ^e^	5.8 ^d^	4.4 ^d^	3.3 ^b^
MRP140	6.0 ^f^	3.3 ^a^	6.9 ^f^	4.3 ^c^	5.6 ^e^	2.7 ^a^

The result of each sensory attribute was listed in means score (n = 45; 15 panelists with 3 replications) and the mean values for each attribute with different letters (a, b, c, d, e and f) were significantly different (*p* < 0.05) using Duncan’s multiple comparison test.
